# Ecosystem Services of Mangrove Forests: Results of a Meta-Analysis of Economic Values

**DOI:** 10.3390/ijerph17165830

**Published:** 2020-08-12

**Authors:** Michael Getzner, Muhammad Shariful Islam

**Affiliations:** Department of Public Finance and Infrastructure Policy, Institute of Spatial Planning, Vienna University of Technology (TU Wien), Karlsplatz 13, 1040 Vienna, Austria; jewelsharif@yahoo.com

**Keywords:** mangrove forests, ecosystem services, environmental health, environmental valuation, meta-analysis, benefit transfer

## Abstract

Mangrove forests are paramount for sustaining and enhancing ecosystem services benefitting both local and regional communities, and the global environment. Scholars have long studied the values of ecosystem services of mangrove forests. However, the number of recent primary studies monetizing ecosystem services is rather limited. This paper ascertains the values of ecosystem services of 66 primary valuation studies with a total of 250 observations. The results indicate that the range of values is substantially wide. This range cannot be explained sufficiently by the various differences of the studies, as the explanatory power of the econometric estimations is low. Main influential factors on the values of ecosystem services are the elicitation methods, the types of ecosystem services considered, and the conservation status of the respective mangrove forest as Ramsar site. The results stress the significant economic values of ecosystem services of mangrove forests and the importance for conservation management. However, the results also caution against a direct transfer of benefits between sites. The substantial variety of site and country specifics warrants the implementation of separate, original valuation studies.

## 1. Introduction

Mangrove forests provide a wide range of ecosystem services. As tropical and subtropical ecosystems, they play a vital role in supporting local and regional coastal communities with ecosystem services (ES) and thus enhancing the livelihoods of communities. Provisioning ecosystem services (e.g., timber and fuel wood), supporting ES (e.g., breeding and nursery habitats for fish species), and regulating ES (e.g., protection from storms and floods, erosion control) are among these locally and regionally important ecosystem services. On a larger scale, regulating carbon and nutrient cycles, as well as cultural ecosystem services, are of global significance (e.g., [1]). Ecosystem services can be defined as the welfare contributions and benefits people derive from ecosystems [2]. Ecosystem services have thus been labeled ‘Nature’s contributions to people’ [3].

The ecological importance of mangrove forest ecosystems originates in the intertidal ecotones of estuaries and open shorelines, and the dynamics of changing water levels, temperatures, erosion and pioneer habitats. These specific conditions result in biodiversity hotspots in only a limited number of countries.

Mangrove forests are under pressure and stress from various directions [4]. The exploitation of resources, sea level rise and climate change, as well as weak governance systems contribute to the degradation of these sensitive ecosystems (e.g., [5]). These circumstances have increasingly reduced the forests’ capacities to sustain ecosystem services for local as well as global communities.

In order to make the economic values of mangrove forests more explicit, the value of ecosystem services of mangrove forests have been determined in environmental valuation studies [6]. Methods of environmental valuation offer a more comprehensive assessment of the various goods and services which may also assist planners, governmental agencies and non-governmental organizations (NGOs) in making decisions about sustainable mangrove forest management [7,8,9].

Moreover, ascertaining the economic value of the ecosystem services of mangrove forests is paramount for evaluating the values that are lost, or will be lost, owing to unsustainable practices and lack of the implementation of consistent national and international conservation policies.

Various studies have investigated the economic value of ecosystem services of mangrove forests. There are revealed and stated preference methods, which assess use and non-use values. International guidance documents and best-practice recommendations are available, e.g., for direct (stated preference) valuation studies [10].

To fully assess all types of values of ecosystem services, there is currently no single method that could deal with all the different kinds of ecosystem services. Instead, a wide range of methods is implemented, which makes comparisons between studies difficult. To explore the differences between studies, meta-analyses have been conducted for environmental valuation objectives.

Applying meta-analyses in the fields of environmental valuation has been popular since the 1990s (e.g., [11,12,13]). Besides solving methodological problems and issues (e.g., how the elicited values are linked to the application of valuation methods), the main aim of meta-analyses is to enhance the knowledge about certain ecosystems in a joint framework. Furthermore, such analyses may also be used for transferring benefits from a ‘study site’ to a ‘policy site’ without the need to conduct primary valuation studies at the policy site (e.g., [14,15]). Assuming that a benefit-transfer function could be estimated with sufficient precision, the attributes of the policy site are used for estimating the values of ecosystem services by means of the benefit-transfer function.

However, there is no recent meta-analysis of environmental valuation studies of ecosystem services in mangrove forests. The aim of this paper is, thus, to collect relevant and recent environmental valuation studies dealing with mangrove forests. Furthermore, the study presents a meta-analysis of the determinants of the economic values of ecosystem services according to international classifications, and combines data from both the scientific studies collected, and official statistics on country-specific variables. Ultimately, if the estimations of the meta-analysis provide useful and comprehensive, statistically robust results, the estimates may also be used for transferring values between sites.

The structure of the paper is as follows: The data and materials used for the analysis are presented in Section 2. Descriptive results are included in Section 3, while econometric results are shown in Section 4. In Section 5 and Section 6, the results are discussed, and conclusions are drawn.

## 2. Materials and Methods

As outlined in the introduction, the aim of the analysis is to ascertain the determinants of economic values of ecosystem services in mangrove forests. The analysis consists of two consecutive steps. First of all, a database was set up that included the economic values of ecosystem services. Selected databases such as Scopus, ScienceDirect or SpringerLink were searched for original valuation studies. The main keywords for the search were combinations of search strings such as “mangrove forests”, “ecosystem services”, “regulating/supporting/provisioning/cultural”, “economic values”, and “environmental valuation”.

The search resulted in 250 original studies (papers, books, theses, and studies of grey literature) to be assessed in more detail. The keywords turned out to be appropriate for setting up a comprehensive database. However, out of 250 original studies, the majority was not included in the final draft because either mangrove forests were only mentioned in the study, and the original values were in fact values transferred from other studies, or basic data on elicitation procedures or ecological and geographical conditions were missing. In addition, the search of empirical studies was limited to research with an explicit focus on mangrove forests. Studies with broader topics such as the valuation of wetlands, rainforests, or other coastal ecosystems, were thus excluded from the analysis.

After a thorough review, in total 66 studies were included (these studies were published in 57 papers or books). Based on these studies, 250 original values of ecosystem services were collected. For each study included, additional data such as variables denoting GDP (Gross Domestic Product), population, inflation (measured by the GDP deflator), were retrieved. In order to facilitate the comparison between the studies, the values of ecosystem services were transformed into normalized values (values of ecosystems, denoted in USD/ha.a at 2018 prices). While it would be straightforward to classify countries according to their ‘status of development’ (e.g., industrial or developing countries), the measure of GDP is – among other indicators – a more detailed and refined indicator for a country’s economic resources to implement effective conservation policies. This paper therefore does not use a simple classification of the ‘status of development’.

Secondly, the analysis consists of descriptive statistics (such as, mean and range values) and econometric estimations (statistical modeling) in order to ascertain the determinants of the values of ecosystem services. For the econometric procedure, standard (weighted) OLS (ordinary least squares) estimations accounting for heteroscedasticity are used.

## 3. Descriptive Results

The following provides an overview of selected key findings of the database. On the one hand, a major issue is which types of ecosystem services are accounted for in each study. Based on the international CICES (cices.eu [16]; Common International Classification of Ecosystem Services) classification, this paper classifies ecosystem services (ES) according to four different categories:

- Provisioning ES (e.g., food, building materials, drinking water);
- Supporting (maintaining) ES (e.g., prevention of coastal erosion);
- Regulating ES (e.g., carbon sequestration, micro climate); and
- Cultural ES (e.g., recreation, spiritual values, biodiversity).

An important attribute for the classification of the studies included in the database is the location of the mangrove forest to be investigated. Table 1 presents an overview of the database, and details the countries, in which mangrove forests were objects of research on the valuation of ecosystem services (detailed references are presented in the reference list: [17,18,19,20,21,22,23,24,25,26,27,28,29,30,31,32,33,34,35,36,37,38,39,40,41,42,43,44,45,46,47,48,49,50,51,52,53,54,55,56,57,58,59,60,61,62,63,64,65,66,67,68,69]). It is not surprising that the number of papers is highest for Asia given that mangrove forests are most prominent in Asian countries such as Indonesia, Malaysia, India and Bangladesh. About two thirds of all the values of ecosystem services in our database originate in Asian mangrove forests. In addition, there are some studies for mangrove forests in Africa, while the database includes only very few papers on forests in America, Europe and Oceania.

In regard to the statistical analysis, it has to be stressed that—as described obove in Section 2—the database was not set up according to some form of random sampling. Papers and studies, and the geographical location of the mangrove forests, were chosen based on their originality and the details provided that were needed for the meta-analysis of this paper. Furthermore, geographical variables denoting the location of the mangrove forests are based on continents rather than single countries owing to the limited number of single-country studies.

Besides the locations of the mangrove forests included in the database, it is of high interest to consider the status of conservation (nature protection) of forests. From a viewpoint of nature conservation, it is interesting to see that the majority of the values of ecosystem services originates from forests that are managed according to some regime of ecological conservation (Table 2). 73.6% of the ES values are connected to areas of nature conservation. The most important category of conservation are national parks and national reserves (some according to IUCN’s (World Conservation Union) management guidelines for national parks), and Ramsar sites (including some that are also listed as World Heritage Site).

In regard to the status of conservation of the mangrove forest for which the database includes at least one value of ecosystem services, it has to be noted that forests may also be developing and changing. Some may be established as protected areas in the future, or may only recently have been restored. Owing to the lack of data, a more detailed differentiation cannot be made. This paper therefore has to stick to the official status of conservation at the time of the publication of the respective valuation study

The descriptive statistical results presented in Table 3 indicate that there is a rather wide range of the values of ecosystem services. Even if normalized to account for different years, currencies and the various sizes of the mangrove forest, the range is huge. On average, the value of ecosystem services of mangrove forests amounts to about 21,100 USD/ha.a (2018 prices). The range of ES values is largest for cultural ecosystem services. Cultural ES (such as the conservation of biodiversity expressed as non-use values) exhibit the greatest mean (about 49,300 USD/ha.a), but also the largest standard deviation. Mangrove forests also provide important regulating ecosystem services (such as carbon sequestration or erosion and flood control), amounting to about 36,100 USD/ha.a. Provisioning and supporting ecosystem services clearly exhibit the smallest values. Interestingly, these ecosystem services are also the most important ones for the livelihood of local residents.

Furthermore, the economic valuation of ecosystem services according to four broad categories of methods exhibits a wide range of values as well (Table 3). Methods of analyzing market prices, for instance, of locally harvested resources, result in lower values, while methods of replacement costs, and travel costs, lead to more substantial values. Methods based on diverse approaches to elicit the local households’ willingness-to-pay (WTP) for the conservation or improvement of the local ecosystem reveal the smallest economic values. The latter result is not surprising, since most mangrove forests are located in countries with low household incomes. However, the standard deviation of values elicited by WTP methods is particularly large, which indicates a great diversity of WTP values.

It is also interesting to note that values for ecosystem services are below average in mangrove forests that are protected.

Finally, Table 3 also shows that the countries where the mangrove forests are located are extremely diverse in terms of the GDP as well as population. The mean income of the countries amounts to about USD 5400 (per capita). However, countries of very low and very high average household income are included. The size of the countries measured by population varies from 0.25 million to over 1.4 billion. A similarly wide range can be observed in regard to the size of the mangrove forest, ranging from 2 to 770,000 hectares.

In summary, these results show that a simple transfer of values (benefit transfer) from one mangrove forest (as a study site where the values are ascertained) to another mangrove forest (as a policy site where the values of the study site are used and transferred to for designing conservation policies without having to conduct primary valuation studies) might be highly problematic. Furthermore, it can be expected that econometric estimations of a benefit transfer function, taking into account various factors that are potentially influential for ES values, might only explain a small part of the variations.

## 4. Econometric Results

In order to estimate a potential benefit-transfer function, the normalized economic values of ecosystem services are treated as the dependent variable in the econometric estimation. Table 4 displays the explanatory variables of the estimations.

The first variable, SIZE, tests for the hypothesis that larger areas may face marginally decreasing money values of ecosystem services. For instance, smaller forests might be more relevant for specific coastal regions, and might therefore exhibit larger per-hectare values.

The country’s gross domestic product (GDP) is included to account for potential influences of the average income of households, and thus mirrors the economic resources and the economic potential to conserve ecosystem services. (Generally, the willingness-to-pay to sustain or improve ecosystem services is positively correlated with income.)

Two variables, ES_SUPP and ES_REG, both account for basic ecosystem services in comparison to the baseline consisting of cultural as well as provisioning ecosystem services.

To reflect the type of publication, the variable PUBL_REV refers to studies that have been peer-reviewed, such as papers published in international peer-reviewed journals. It can be hypothesized that peer-reviewed papers provide more robust values of ecosystem services because the methodology is reviewed in the peer review process.

To account for the methodological differences of the various studies, the variables METH_RC, METH_TCM, and METH_WTP, denote the environmental valuation technique that was primarily used for ascertaining the values of ecosystem services.

In order to reflect the geographical focus of the studies, we differentiate between studies dealing with mangrove forests in Africa and Oceania (compared to the baseline of Asia, America, and Europe).

Finally, the conservation status is also taken into account to examine differences between forests that are protected and forests that are not protected.

Table 5 presents four estimations that are different in regard to the explanatory variables included.

Est. 1 presents a basic econometric estimation in which the conservation status as well as a more detailed geographical explanation are omitted. First of all, it seems that the SIZE variable does not contribute to the explanatory power of the model. In all estimations, the coefficient of this variable is far from being significant.

Secondly, the GDP variable only exhibits a modest influence on the values of ecosystem services. The coefficient of this variable is close to being insignificant for the Est. 1, but definitely exhibits a larger explanatory power in Est. 4.

As expected by analyzing the descriptive evidence presented in Section 3 above, values of supporting ecosystem services are significantly smaller than the baseline ES, while regulating ES are correlated with higher economic values of ecosystem services. This effect can be seen in all four estimations (Est. 1 to 4).

The results for the coefficient of the variable PUBL_REV are in line with previous expectations. The values of ecosystem services published in peer-reviewed papers are—ceteris paribus—smaller than the values presented in other studies.

In turn, the methods applied for the environmental valuation of ecosystem services indicate that the elicitation methods clearly have a significant effect on the dependent variable. Compared to the baseline of market prices, the different methods yield larger ES values, all other influences held constant.

Finally, the variable AFRICA, which is included in Est. 1, exhibits a weakly significant coefficient. The resulting adj. R² of the estimation is low and amounts to about 13.5% of the variance of the dependent variable.

In order to further investigate the significance of explanatory variables, Est. 2 is a variant of Est. 1 that adds the variable PROTECTED to the econometric estimation. The statistical quality of the estimation remains in the same order of magnitude. Interestingly, this variable does not seem to influence the dependent variable.

In Est. 3, the variable PA_RAMSAR is included. This variable account for the status of conservation of the mangrove forest as a Ramsar site (or a combined Ramsar and World Heritage site). In the process of estimating the most appropriate model, several attempts were made in regard to various variables denoting different types of conservation. The inclusion of the variable PA_RAMSAR finally proved to be most successful. The change of some variables definitely improved the statistical quality of the estimation with an enhanced adj. R²-value of 19.4%.

Finally, the Est. 4 is the estimation with the highest statistical fit. Along with exchanging the variable AFRICA by the variable OCEANIA, and by including the status of conservation (variable PA_RAMSAR), the adj. R² is improved to 24.4%. However, the variable PUBL_REV does not exhibit a significant coefficient any more, while the other coefficients stay roughly in the same order of magnitude.

## 5. Discussion

This paper is based on the assumption that two groups of factors can explain the variances among the economic values of ecosystem services. The first group of variables is independent of the valuation study investigated. Variables of this first group contain, for instance, the size of area, the status of conservation, and the country’s population and GDP. The second group of variables is inherent to the respective valuation study. For instance, this group includes the methodology of the valuation study, or the process of publication.

In general, similar meta-analyses in the literature exhibit a greater statistical fit than the estimations presented above. Especially for the specific ecosystem of mangrove forests, it seems that such common ground of valuation studies cannot be found easily. The statistical analysis can only explain about one quarter of the variances between the economic values of ecosystem services. As already highlighted in the descriptive analysis, the contexts and country specifics are too varied to allow for a joint analysis (cf. [70]).

However, it is a well-known problem of meta-analyses such as the one presented here that the existing empirical studies are usually conducted with a specific empirical focus, and within a specific policy framework. In addition, a different methodological expertise of both researchers and commissioning institutions leads to mixed applications of the valuation methods.

While the explanatory power of the estimations presented above are limited, some a priori hypotheses can be confirmed. For instance, the values published in peer-reviewed papers are smaller than in other publication outlets, and different valuation methods may alter the valuation results. Having said this, it should also be noted that the different valuation methods measure a wide range of benefit categories (e.g., use and non-use values). Furthermore, the econometric analyses revealed that mangrove forests, which are protected under the most prominent international framework of the conservation of wetlands, the Ramsar Convention, provide significantly greater values of ecosystem services to the local and regional communities. However, the approach used in this paper cannot determine whether or not there is a causal relationship between the declaration of protected areas as Ramsar sites, and the values of ecosystem services. It can certainly be assumed that forests of the most ecological value (e.g., pristine forests) are more likely to be chosen as Ramsar sites. One should, though, not forget that conservation policies and decisions are also subject to political and economic reasoning, which might lead to sub-optimal conservation compared to the ecologically best option.

Besides the status of conservation as a Ramsar site, mangrove forests are constantly changing in regard to their ecological dynamics. Therefore, this paper relies on the status of conservation at the time when the underlying valuation study was published. For future research, it would be promising to assess the values of ecosystem services depending on a more detailed evaluation of the status of conservation. For instance, ecosystem services might not be significantly different between areas of substantial ecological values, but which are not yet protected under some legal framework, and those, which are already conserved.

Furthermore, it is not only the specific focus and context of each of the studies that reduce the validity of the results, but also the comparatively limited number of original studies that could be utilized for the analyses of this paper. Of course, there are numerous environmental valuation studies available for all types of ecosystem services (see, e.g., [71]). However, in order to provide answers to the research questions, publications had to meet certain criteria (e.g., inclusion of critical information) so that they could be used for the database of this paper.

The empirical values of ecosystem services in the database underlying this paper exhibit a large range. However, it has to be noted that some outliers distort the mean values. These outliers basically refer to a few papers in which two kinds of ecosystem services were valued with substantial amounts. One group includes ecosystem services protecting agricultural and residential areas from coastal erosion; the other one accounts for the recreation function of mangrove forests (especially international tourism). One has therefore be careful in drawing quick conclusions since these results provide an additional argument for a detailed and site-specific analysis instead of taking mean values for transferring benefits between sites.

## 6. Conclusions

This paper has provided an overview of the economic (monetary) values of ecosystem services of mangrove forests in the framework of an econometric meta-analysis. The available studies highlight the importance of mangrove forests for a rather small range of countries primarily in Asia. The ecosystem services of mangrove forests are substantial; the scientific studies reviewed in this paper specifically address regulating and provisioning ecosystem services, which are particularly important for the wellbeing and for the livelihoods of local residents. Globally important values such cultural ecosystem services, which are usually of great importance in valuation studies of other types of ecosystems, are of less significance in the empirical literature concerning mangrove forests. As such, the results of this study support the recent conclusions drawn by a review of empirical papers on coastal wetlands in regard to the need of more empirical studies on economic values of wetlands [72].

The ultimate objective of this paper was to estimate a benefit-transfer function for the values of ecosystem services that could be used to determine the values in other locations or different contexts. This goal could only partially be achieved. The results suggest that the explanatory power of the statistical models is limited. The models could explain only up to about a quarter of the variances in the values of ecosystem services. The estimates thus cannot be used for a direct transfer of benefits from one site to another.

In regard to the method of estimating the values of ecosystem services, the results of the paper indicate that the choice of the methods matter for the estimations. Not all types of ecosystem services can be valued by the most ‘conservative’ methods valuation such as deriving values from market prices. For many ecosystem services, markets simply do not exist. However, it can be concluded that—for policy purposes in the context of benefit-cost analysis—methods should be chosen that reveal robust environmental values.

The results of this paper, though, highlight the eminent importance of the values of ecosystem services of mangrove forests. In addition, the estimations also suggest that strict conservation policies such as the Ramsar convention are closely linked to the provision of ecosystem services, and that the interlinkages between conservation planning, management and governance system become evident. For some mangrove forests, studies have shown that strong governance systems can significantly contribute to the benefits of ecosystem services for the local communities ([73]). The results of this paper therefore also stress the economic value of such conservation and governance regimes. The discussion of the results has emphasized that the merely legal perspective on the status of conservation might be insufficient to assess the values of the ecosystem services of the mangrove forests. However, as this paper has also shown the limited availability of studies comprehensively valuing the ecosystem services of mangrove forests in more standardized methodological frameworks, future research on the environmental valuation of these globally important ecosystems should follow the international guidance documents such as Johnston et al. [10] for revealed and stated preference elicitation methods.

## Figures and Tables

**Table 1:** Distribution of the values of ecosystem services included in the database.

Continent	Country	References	No. of Values of Ecosystem Services
Africa	Kenya, Nigeria, Mozambique, Egypt	[18,19,30,37,48,57,67,69]	56
America	Belize, Colombia, Brazil, Mexico, Guyana, Bahamas, Ecuador	[1,32,50,51,55,60,63,64]	16
Asia	Indonesia, Vietnam, Sri Lanka, Thailand, Philippines, Myanmar, Malaysia, Pakistan, India, China, Bangladesh	[7,8,17,20,21,22,23,24,25,27,28,29,30,33,35,36,38,39,40,41,42,43,44,45,46,47,49,52,53,54,56,58,59,61,62,65,66,68]	165
Europe	France	[34]	2
Oceania	Vanuatu, Fiji	[26,31]	11
Total no. of observations (values)			250

Source: Own compilation and database, 2020.

**Table 2:** Selected descriptive statistics of the papers and studies collected for the database referring to the conservation status of the considered mangrove forests.

Variables of Conservation Status	Frequency
Values of ecosystem services of mangrove forests not protected	66
Values of ecosystem services of mangrove forests protected,	184
of which:	
Ramsar Sites (including combinations with World Heritage Sites)	41
Biosphere Reserves	21
National parks (including combinations with World Heritage Sites, National Protected Areas, National Reserves)	67
Nature Reserve (including Sanctuaries and Reserve Forests)	29
Marine Protected Area (including Marine National Parks, Marine Nature Parks)	28
Total (no. of observations)	250

Source: Own compilation and database, 2020.

**Table 3:** Descriptive statistics of the values of ecosystem services (ES) of mangrove forests.

Variables		Unit of Measurement	Mean	Std.Dev.	Min.	Max.	Observations
*Values of the ecosystem services (ES) of the respective mangrove forest*							
All ES values (full sample)		USD/ha.a, 2018 prices	21,071.81	132,705.50	0.52	1,432,142.00	250
Values of ES in regard to the type of ES	Provisioning		4897.79	16,951.90	0.52	154,645.60	105
	Regulating		36,100.91	172,911.19	1.28	1,395,925.74	74
	Supporting		401.68	739.27	7.92	3183.11	29
	Cultural		49,299.21	225,375.70	0.69	1,432,142.00	42
Values of ES in regard to the valuation method	Market prices		9008.47	41,333.01	0.52	464,431.50	178
	Replacement costs		93,370.69	329,606.64	6.07	1,395,925.74	18
	Travel costs		94,164.08	324,040.33	12.44	1,432,142.00	20
	Willingness-to-pay		2955.58	9139.77	3.46	41,394.37	34
Values of ES of mangrove forests that are protected			17,718.40	114,638.46	0.52	1,432,142.00	184
Gross domestic product (GDP)		USD (per capita, 2018 prices)	5366.77	6290.09	490.00	41,464.00	66
Population (POP)		Residents (million)	200.65	314.54	0.25	1414.05	66
Size of the mangrove forest (SIZE)		Hectares (ha)	57,158.98	174,601.16	2.00	770,000.00	66

Source: Own calculations, 2020.

**Table 4:** Description of the dependent and explanatory variables of the estimations of the meta-analysis.

Variables	Description
*Dependent variable*	
VALUE	Value of ecosystem service (in ln USD per ha.a, 2018 prices)
*Explanatory variable*	
SIZE	Size of the mangrove forest (in ln hectares)
GDP	Gross domestic product of the country where the respective mangrove forest is located (GDP, in ln USD per capita, 2018 prices), as an indicator of the economic resources of a country to implement effective conservation policies
ES_SUPP	=1 for supporting ecosystem services
ES_REG	=1 for regulating ecosystem services
PUBL_REV	=1 for values published in peer-reviewed studies (papers in peer-reviewed journals)
METH_RC	=1 for values based on replacement costs
METH_TCM	=1 for values based on travel cost models
METH_WTP	=1 for values based on studies eliciting willingness-to-pay (e.g., contingent valuation, choice experiment)
AFRICA	=1 for values of mangrove forests in Africa
OCEANIA	=1 for values of mangrove forests in Oceania
PROTECTED	=1 for mangrove forests that are protected
PA_RAMSAR	=1 for mangrove forests that are protected as Ramsar sites (including sites that are Ramsar and World Heritage site)

Source: Own concept, 2020.

**Table 5:** Econometric estimations of the determinants of the values of ecosystem services (benefit-transfer functions).

Variables	Est. 1			Est. 2			Est. 3			Est. 4		
	Coeff.	t-Stat.	Pr.	Coeff.	t-Stat.	Pr.	Coeff.	t-Stat.	Pr.	Coeff.	t-Stat.	Pr.
Constant	2.316	0.872		2.410	0.907		3.707	1.439		0.182	0.079	
SIZE	−0.032	−0.540		−0.018	−0.307		0.012	0.211		0.030	0.541	
GDP	0.509	1.714	*	0.518	1.752	*	0.359	1.264		0.685	2.620	***
ES_SUPP	−1.293	−2.934	***	−1.290	−2.899	***	−1.200	−2.703	***	−1.152	−2.586	**
ES_REG	0.951	2.371	**	0.942	2.347	**	0.992	2.598	***	0.776	2.180	**
PUBL_REV	−0.87	−2.221	**	−0.896	−2.275	**	−1.117	−3.024	***	−0.416	−1.253	
METH_RC	1.284	1.746	*	1.309	1.785	*	1.399	2.122	**	1.584	2.423	**
METH_TCM	2.004	3.335	***	2.029	3.333	***	1.856	2.983	***	1.589	2.454	**
METH_WTP	0.988	1.983	**	1.004	1.995	**	1.325	2.476	**	0.795	1.729	*
AFRICA	−0.971	−1.740	*	−0.955	−1.712	*	−1.212	−2.209	**			
OCEANIA										3.577	5.068	***
PROTECTED				−0.336	−0.838							
PA_RAMSAR							−1.945	−4.547	***	−1.632	−3.974	***
Adj. R²	0.135			0.134			0.194			0.244		
S.E. of regr.	2.585			2.586			2.495			2.416		
Log likelihood	−587.041			−586.663			−577.717			−569.688		
F-statistic	5.310 ***			4.846 ***			6.978 ***			9.027 ***		
N (studies)	66			66			66			66		
n (obs.)	250			250			250			250		

Notes: Dependent variable: VALUE; estimation: Weighted LS (least squares) estimation with Huber–White–Hinkley (HC1) heteroskedasticity, consistent standard errors, and covariance; *** *p* < 0.01; ** *p* < 0.05; * *p* < 0.1. Source: Own calculations, 2020.
